# *SMPD3*‐*ALK*: A novel *ALK* fusion gene in lung adenocarcinoma

**DOI:** 10.1111/cge.13891

**Published:** 2021-02-08

**Authors:** Yuepei Liang, Yang Wang, Wenjing Wang, Juan Zhao, Mian Xu, Min Zheng

**Affiliations:** ^1^ Department of Thoracic Surgery Affiliated Hospital of Guilin Medical University Guilin China; ^2^ OrigiMed Shanghai China

## Abstract

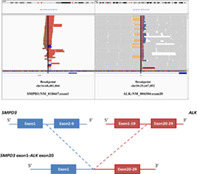

## PEER REVIEW

The peer review history for this article is available at https://publons.com/publon/10.1111/cge.13891.

Anaplastic lymphoma kinase (*ALK*) is one of the most important driver genes and therapeutic targets in patients with non‐small‐cell lung cancer (NSCLC), who tend to be young and either light smokers or nonsmokers.^1^ Next‐generation sequencing (NGS) technology, which is widely used in clinical practice, allows clinicians to identify both the 5′ partner and 3′ kinase involved in *ALK* fusions. Various ALK fusion partners have been identified in NSCLC, which are associated with the response to ALK tyrosine kinase inhibitors.^2^, ^3^ Here, we report a case of lung adenocarcinoma harboring a novel *SMPD3*‐*ALK* fusion gene.

**FIGURE 1:**
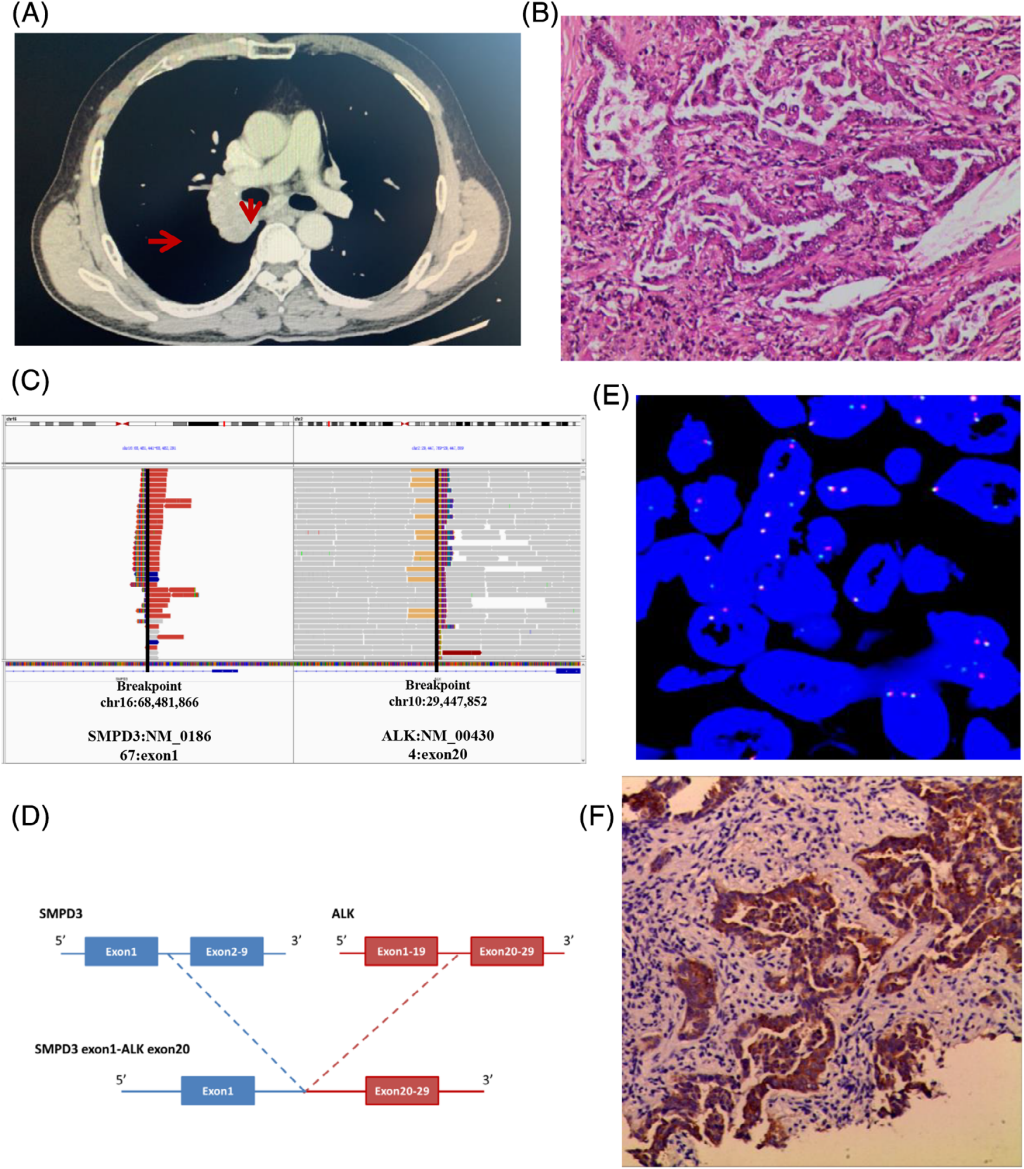
Lung adenocarcinoma identified by CT scan and HE staining and illustration and verification of the *SMPD3‐ALK* fusion. (A) Chest CT scan shows a mass in the right hilum with mediastinal lymph node metastasis (arrow). (B) HE staining of the patient (original magnification ×100). (C) NGS results showing the breakpoint of *SMPD3‐ALK* fusion. (D) Diagrammatic sketch of *ALK* fusion. (E) FISH staining verified the *SMPD3‐ALK* fusion (original magnification ×1000). (F) Immunohistochemical staining reveals *ALK* expression (original magnification ×200). ALK, anaplastic lymphoma kinase; CT, computed tomography; HE, hematoxylin and eosin; NGS, next‐generation sequencing; SMPD3, sphingomyelin phosphodiesterase 3 [Colour figure can be viewed at wileyonlinelibrary.com]

A 56‐year‐old nonsmoking Chinese male was admitted to our hospital with cough. A chest computed tomography (CT) scan revealed a 4.4 × 3.3 cm^2^ mass in the right hilum, with mediastinal lymph node metastasis (Figure 1(A)). Stage IIIA lung adenocarcinoma was confirmed based on CT‐guided lung puncture pathology (Figure 1(B)). To determine potential therapeutic regimens, the tumor sample was sent for NGS analysis using a DNA panel of 450 cancer‐related genes. Informed consent was obtained from the patient. *SMPD3*‐*ALK*, a fusion generated from the fusion of exon 1 of *SMPD3* and Exons 20–29 of *ALK* (Figure 1(C),(D)), was identified, and verified by FISH assay (Figure 1(E)). COSMIC fusion databases (https://cancer.sanger.ac.uk/cosmic/fusion) and Quiver fusion databases (http://quiver.archerdx.com/) confirmed that the *SMPD3*‐*ALK* fusion identified in this case is a novel fusion.

Sphingomyelin phosphodiesterase 3 (SMPD3), an enzyme encoded by *SMPD3* in humans, is involved in the pathway sphingolipid metabolism. It also may has cellular response to tumor necrosis factor (GO:0071356). A genome‐wide study has shown that *SMPD3* is a potential repressor of hepatocellular carcinoma, playing an important role in tumor formation.^4^ Here, the breakpoints of *SMPD3‐ALK* fusion were located in the Intron 1 of *SMPD3* and the Intron 19 of *ALK* that preserves the intact kinase domain of the ALK and may lead to the activation of ALK kinase. Similarly, *EML4‐ALK* fusion with similar breakpoints occurring in the Intron 19 of *ALK* activates the downstream RAS/MAPK, PI3K/Akt, and JAK signaling pathways.^5^ Here, the activation of ALK was confirmed by immunohistochemistry (Figure 1(F)).

To date, crizotinib, ceritinib, alectinib, and brigatinib have been approved for the treatment of *ALK* fusion NSCLC. It has been shown that sequential use of ALK inhibitors may clinically benefit patients showing progress on an initial ALK inhibitor.^6^ Therefore, the use of ALK inhibitors in the later stages of treatment might be effective in our patient, who underwent surgical resection and received adjuvant chemotherapy post‐operatively and no recurrence has been observed so far. However, future studies comparing the efficacy of ALK inhibitors against different variants of NSCLC are warranted.

In conclusion, we present the first report of *SMPD3*‐*ALK* fusion, which will expand the spectrum of known *ALK* fusion variants. By broadening the understanding of *ALK* fusions, our case study will help clinicians improve the precision of patient care.

## CONFLICT OF INTEREST

Juan Zhao, Mian Xu and Wenjing Wang received personal fees from OrigiMed; the remaining authors declare no potential conflict of interest.

## Data Availability

Data sharing is not applicable to this article as no new data were created or analyzed in this study.
